# Resolving and Controlling Silicoaluminophosphate Zeolite Intergrowths and Mixtures

**DOI:** 10.1002/advs.76192

**Published:** 2026-06-19

**Authors:** Yuxin Ke, Jiale Feng, Lei Wang, Xiao Chen, Mengmeng Ma, Jiabin Cui, Juan Diwu, Yuman Liu, Fei Wei, Boyuan Shen, Bin Song

**Affiliations:** ^1^ Institute of Functional Nano & Soft Materials (FUNSOM) Soochow University Suzhou Jiangsu P. R. China; ^2^ Jiangsu Key Laboratory of Advanced Negative Carbon Technologies Soochow University Suzhou Jiangsu P. R. China; ^3^ Beijing Key Laboratory of Green Chemical Reaction Engineering and Technology, Department of Chemical Engineering Tsinghua University Beijing P. R. China; ^4^ State Key Laboratory of Radiation Medicine and Protection, School of Radiation Medicine and Protection, Collaborative Innovation Center of Radiological Medicine of Jiangsu Higher Education Institutions Soochow University Suzhou Jiangsu P. R. China; ^5^ Suzhou National Laboratory Suzhou Jiangsu P. R. China

**Keywords:** atomic imaging, controlled synthesis, electron microscopy, intergrowth, silicoaluminophosphate zeolite, X‐ray diffraction

## Abstract

Achieving controlled synthesis of silicoaluminophosphate (SAPO) zeolites is critical for optimizing their catalytic performances, yet it remains difficult due to the complex coupling of synthesis variables and insufficient atomic‐level structural understanding. Herein, we systematically investigate the multi‐scale characterization and controllable synthesis of SAPO zeolites. We explore the effects of critical synthesis parameters (pH, Si content, the concentration and composition ratio of structure‐directing agents) on the structural selectivity and distribution of SAPO‐5 (AFI) and SAPO‐34/18 (CHA/AEI) intergrowths and mixtures, identify the selective synthesis window for each structural component, and interpret the underlying selectivity mechanism from a perspective of competitive thermodynamics and kinetics. Low‐dose electron microscopy enables the atomic‐resolution imaging of beam‐sensitive SAPO‐34/18 intergrowths for revealing their stacking sequences and spatial distributions under the regulation of Si content and structure‐directing agent ratio. This work provides a methodology for investigating the structure‐property relationship of zeolite structures by combining controllable synthesis with atomic‐resolution electron microscopy, thereby enabling the controllable design of these zeolite catalysts with tailored topology and porous structure.

## Graphical abstract

Low‐dose electron microscopy enables atomic‐resolution imaging of beam‐sensitive SAPO‐34/18 intergrowths, revealing their stacking sequences and spatial distributions under controlled Si content and SDA ratio. This work establishes a methodology linking controllable synthesis with atomic‐resolution microscopy to investigate structure–property relationships, thereby enabling the rational design of zeolite catalysts with tailored topology and porous structure.

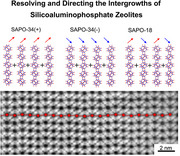

## 1 Introduction

The differences and similarities in dynamics and thermodynamics between the target material and its competitors are of great significance for their controllable synthesis and function design [1, 2, 3, 4]. In complex synthesis environments, small changes in conditions can lead to great differences in some structural characteristics such as atomic arrangement, elemental composition, and spatial topology of the material product, thereby affecting its performance [5, 6].The silicoaluminophosphate (SAPO) family is typical example in zeolite synthesis, where several tens of framework types (three‐letter codes) have been reported based on Si, Al, P, O elements [7]. In these zeolite structures, SAPO‐34 (CHA) and SAPO‐18 (AEI) were widely used as catalysts in the methanol‐to‐olefins (MTO) process [8, 9, 10, 11], one of the most successful industrial applications of zeolite catalysis, enabling the production of light olefins from non‐petroleum feedstocks, while their competitor SAPO‐5 (AFI) is ineffective for MTO [7, 12]. The SAPO‐34, with 7.4 Å cages interconnected by 3.8 Å windows, exhibits exceptional shape selectivity toward ethylene and propylene due to the size matching between the small windows and the molecules, but suffers from rapid deactivation by coke deposition [13, 14]. The SAPO‐18 shares nearly identical cage dimensions with SAPO‐34 but differs in framework connectivity, offering complementary catalytic behavior [15, 16]. On the contrary, the SAPO‐5 has only 7.3 Å straight channels and cannot demonstrate shape selectivity for olefins in catalysis, but it may be obtained with the SAPO‐34 and SAPO‐18 in many synthesis environments [17, 18].

Moreover, the SAPO‐34 and SAPO‐18 share the same primary (SiO_4_, AlO_4_, PO_4_ tetrahedra) and secondary (double six‐membered rings) building units (only different in their spatial arrangement), making the SAPO‐34/18 intergrowths very feasible to form when the common structure‐directing agents (SDAs) such as triethylamine (TEA) and tetraethylammonium hydroxide (TEAOH) are employed [12, 19, 20, 21, 22, 23, 24]. Recent studies [22, 23, 25] have demonstrated that the SAPO‐34/18 intergrowth catalysts can substantially enhance the lifetime in the methanol‐to‐olefins process [8]. Despite the potential performance of these intergrowths, their controlled synthesis is still challenging due to the complex interaction between thermodynamic and kinetic factors governing topology selectivity [26, 27, 28]. Conventional synthesis studies lack a predictive system that correlates different synthesis parameters, including pH, silicon content, and template composition, with the resulting intergrowth architecture [29, 30, 31, 32]. Meanwhile, conventional characterization techniques, primarily based on powder X‐ray diffraction (PXRD), provide only averaged structural information and fail to resolve the local distribution of CHA and AEI domains within intergrowths, which also limit the basic understanding of how synthesis conditions modulate the nanoscale stacking sequences of intergrowths that ultimately determine macroscopic catalytic performance. Recent advances in electron microscopy, particularly the development of integrated differential phase‐contrast scanning transmission electron microscopy (iDPC‐STEM), have opened new opportunities for imaging electron‐beam‐sensitive materials with atomic precision [33, 34, 35, 36, 37, 38, 39]. By enabling direct visualization of light elements within zeolite frameworks while minimizing beam damage [37, 40, 41], iDPC‐STEM offers the potential to resolve the intricate interfacial structures and stacking sequences that define SAPO‐34/18 intergrowths in real space [25, 42].

In this work, we report an in‐depth study on the controllable synthesis and characterization of a series of zeolite structures, focusing on SAPO‐34/18 intergrowths and SAPO‐5 mixtures with CHA, AEI and AFI frameworks, respectively. We systematically investigate how synthesis parameters—including gel pH, Si content, and the total SDA concentration and relative molar ratio of SDAs—govern the products of the hydrothermal crystallization process. Using the PXRD, we quantify the relative fractions of SAPO‐34/18 and SAPO‐5 via their distinct characteristic diffraction peaks, and locate a robust window for synthesizing SAPO‐34/18 intergrowths free of SAPO‐5 byproducts, with the underlying phase control mechanism rationalized by the competitive thermodynamic and kinetic preferences during crystal nucleation and growth. For the fine SAPO‐34/18 intergrowth structures, low‐dose iDPC‐STEM achieves atomic‐resolution imaging of nanoscale alternating SAPO‐34/18 domains and diverse stacking sequences of layered units, providing unprecedented insights into the assembly mechanism of intergrowth zeolite materials under different dual SDA ratios. This work provides a case study for controlling and characterizing the composition and spatial distribution in zeolite intergrowths, laying a methodological foundation for the rational design of SAPO structures with tailored porous properties.

## 2 Results and Discussion

### 2.1 SAPO‐34/18 Intergrowths and SAPO‐5 Mixtures

**FIGURE 1:**
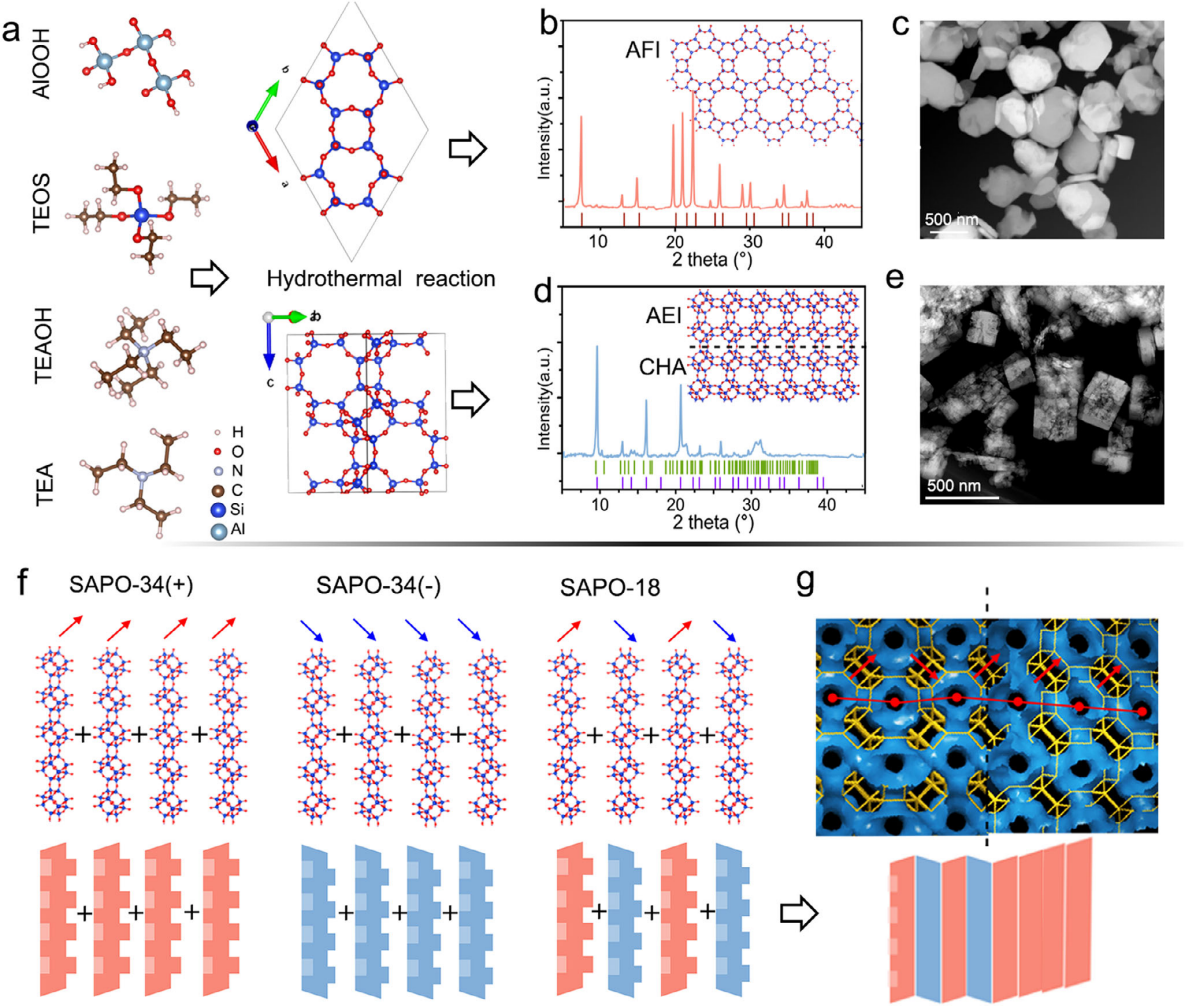
Synthesis and structural model of different SAPO zeolites. (a) Schematic illustration of the SAPO synthesis method. (b, c) PXRD pattern and HAADF‐STEM image of the SAPO‐5 with the AFI framework. (d, e) PXRD pattern and HAADF‐STEM image of the SAPO‐34/18 intergrowth with CHA and AEI frameworks. (f, g) Structural models of the SAPO‐34 (±), SAPO‐18, and their intergrowth based on the layered units with different stacking sequences.

In the synthesis of SAPO zeolites, the primary precursors typically include pseudoboehmite (as the aluminum source), phosphoric acid (as the phosphorus source), tetraethyl orthosilicate (as the silicon source), and SDAs (i.e., TEA and TEAOH). The SDAs play multiple roles in the synthesis process [18, 25], including directing framework formation, compensating for negative charges within the zeolite framework, and functioning as space‐filling species [27], as illustrated schematically in Figure 1a. Notably, under specific synthetic conditions (see details in Materials and Methods and Table S1), we successfully obtained pure SAPO‐5 zeolite with one‐dimensional 12‐membered‐ring channels. As shown in the PXRD patterns in Figure 1b, the observed diffraction peaks at 7.4°, 12.9°, 14.8°, 19.7°, 21.1°, 22.3°, 29.0°, and 29.9° indicate the AFI framework topology, confirming the formation of pure SAPO‐5 structure. The hexagonal prismatic shape of nanocrystals observed in the high‐angle annular dark‐field scanning transmission electron microscopic (HAADF‐STEM) image (Figure 1c) further conforms to the lattice symmetry of AFI framework, as shown in the structural models in Figure 1a,b (see additional characterization in Figures S1 and S2).

In contrast to the SAPO‐5, the CHA‐type SAPO‐34 and AEI‐type SAPO‐18 zeolites consist of interconnected cages with a cage diameter of 7.4 Å and opening windows of only 3.8 Å, and can match each other in a certain crystallographic direction to form their intergrowth structures. Indeed, the PXRD pattern of the as‐prepared SAPO‐34 sample shown in Figure 1d shows obvious shoulder peaks near 10.4° and 21°, suggesting the presence of SAPO‐18 domains within the intergrowth structure. The HAADF‐STEM image in Figure 1e predominantly indicates a near cubic shape of nanocrystals, mainly consistent with the lattice symmetry of AEI framework, as shown in the structural models in Figure 1a,b. In the context of SAPO‐34/18 intergrowth, the two zeolite frameworks can be abstractly described as layered architectures formed by layered building units that stack sequentially along a specific crystallographic direction. As illustrated in Figure 1f, we defined the red units marked by the red units are marked by red arrows pointing diagonally upward from left to right, and the blue units are marked by blue arrows pointing diagonally downward from left to right. Then, the homo‐directional stacking of the red or blue units represents the characteristic of the CHA‐type SAPO‐34 lattice (+ and − is equivalent but rotated 180 degrees), and the hetero‐directional stacking of the red and blue units results in the formation of the AEI‐type SAPO‐18 lattice, just like building Lego blocks. Consequently, the coexistence and certain arrangement of these two stacking modes within the same nanocrystal led to various SAPO‐34/18 intergrowths with alternating CHA and AEI topologies, as schematically depicted in Figure 1g.

### 2.2 Controlling the SAPO Zeolite Structures

To systematically elucidate the influence of key synthetic parameters on the structure selectivity between SAPO‐5, SAPO‐34, and SAPO‐18, we investigated the effects of reaction pH, Si/Al ratio (Si source concentration), total SDA concentration, and the compositional ratio of the TEA/TEAOH binary SDA under single‐factor or multi‐factor controlled conditions. In single‐factor studies, we take the pH parameters as an example. The pH of the synthesis gel, reflecting the acidity or basicity of the reaction medium, emerges as a dominant factor governing the competitive formation of the SAPO‐5, SAPO‐34, and SAPO‐18 structures. Specifically, the pH modulates the solubility of Si, Al, and P precursors, thereby influencing the collision kinetics of reactive intermediates, the assembly of secondary building units, and the degree of Si incorporation into the SAPO framework in the later stages of hydrothermal crystallization. The latter directly dictates the density, strength, and distribution of Brønsted acid sites in the final crystalline material, the key descriptor governing its catalytic performance. Notably, the two SDAs (TEAOH and TEA) employed herein exhibit intrinsic alkalinity upon aqueous dissolution, establishing an inherent coupling between the SDA formulation (total concentration and TEAOH/TEA ratio) and the pH of the initial synthesis gel. Any modulation of the SDA system will therefore inevitably alter the pH environment of the entire synthetic system. Accordingly, to decouple the intrinsic effect of pH on crystallization from the variations in template formulation, we maintained all other interrelated parameters invariant while systematically tuning the pH of the initial synthesis gel across eight discrete values from 5.00 to 8.14. The composition of each as‐prepared sample was semi‐quantitatively evaluated by tracking the intensity change in the characteristic diffraction peaks in PXRD patterns.

**FIGURE 2:**
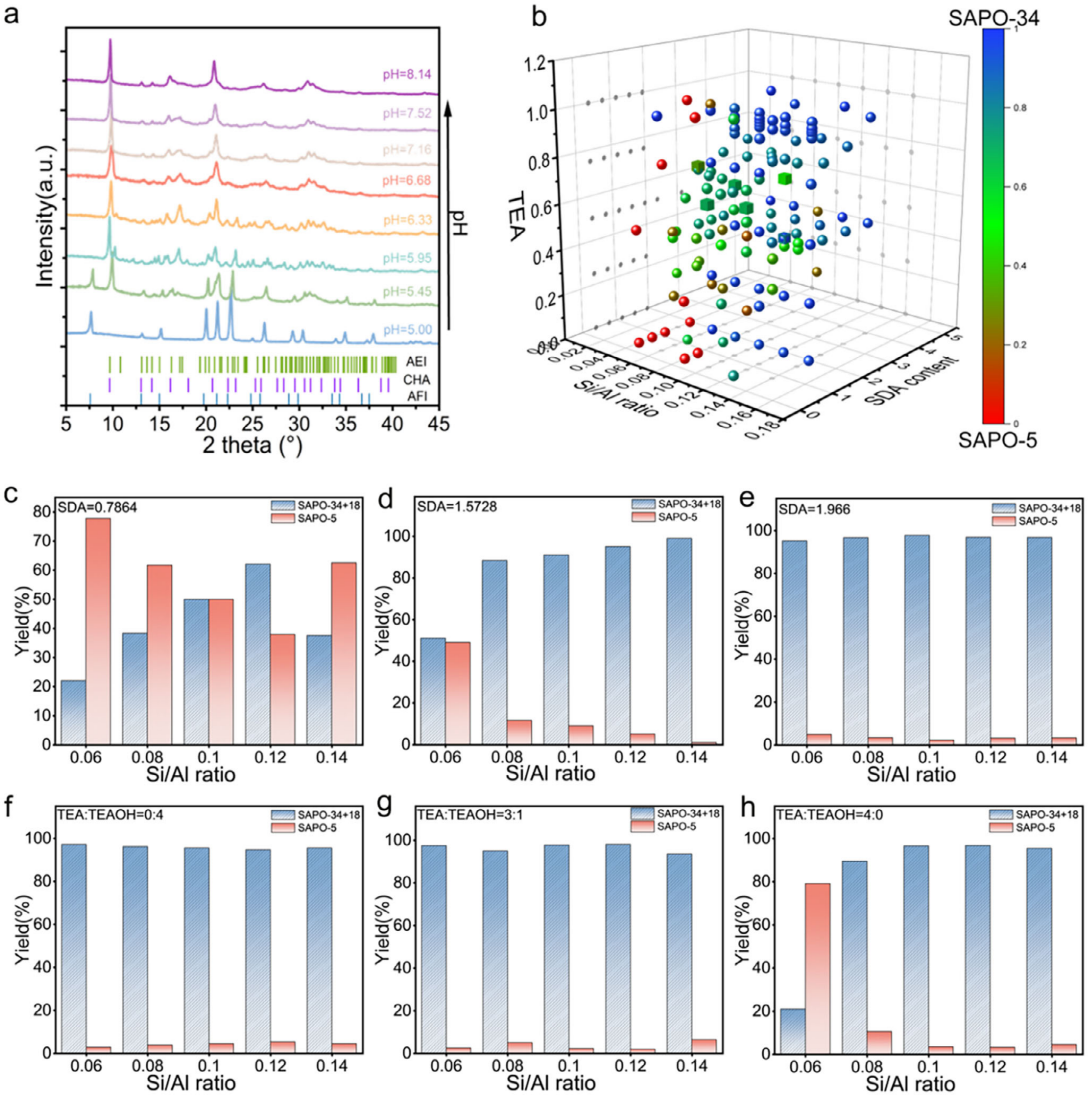
Controlled synthesis and structure analysis of SAPO zeolites based on PXRD results. (a) PXRD results of the SAPO zeolites synthesized in the precursor solutions with different pH values (single‐factor study). (b) Semi‐quantitative PXRD peak analysis for the proportion of SAPO‐5 and SAPO‐34 (and SAPO‐18) in as‐prepared zeolite mixtures in three‐factor studies: Si/Al ratio, total SDA concentration, and dual SDA (TEA/TEAOH) ratio. (c–e) Semi‐quantitative PXRD peak analysis for the proportion of SAPO‐5 and SAPO‐34 (and SAPO‐18) in the as‐prepared zeolite mixtures in two‐factor studies: Si/Al ratio and total SDA concentration. (f‐h) Semi‐quantitative PXRD peak analysis for the proportion of SAPO‐5 and SAPO‐34 (and SAPO‐18) in the as‐prepared zeolite mixtures in two‐factor studies: Si/Al ratio and dual SDA (TEA/TEAOH) ratio.

As shown in Figure 2a, at pH  5.00, the synthesized product consists exclusively of the AFI‐type SAPO‑5. Upon increasing the pH to 5.45, the intensity of the AFI‑characteristic peak (e.g., at 7.4° 2θ) diminishes, while the emergence of a reflection at 9.7° 2θ indicates the incipient formation of the CHA framework. Notably, no reflection at 10.4° 2θ that is attributable to the AEI framework can be detected at this stage. At the pH values of 5.95 and 6.33, the AFI‐characteristic peaks completely disappear, whereas both the reflections at 9.7° and 10.4° 2θ become clearly visible. This observation marks the beginning of AEI formation, leading to a mixed CHA/AEI lattice as the SAPO‐34/18 intergrowth. Further increase of pH to the range from 6.68 to 8.14 results in the gradual attenuation of the AEI‑characteristic peak at 10.4° 2θ, and the PXRD patterns eventually show the reflections of the CHA lattice, the intensities of which progressively increase with the rising pH. Therefore, these results demonstrate a product change with increasing pH, where the composition of mixtures and intergrowths transitions from primarily AFI structure through the CHA/AEI intergrowth, finally to a CHA‑dominant structure as the synthesis gel becomes more alkaline.

Excluding the influence of pH, the Si content (expressed by the Si/Al ratio) and SDA content (including the total SDA concentration and TEA/TEAOH ratio) are also considered as key factors for controlling the SAPO zeolite composition. Similarly, the single‐factor studies of their influences on the SAPO zeolite synthesis were shown by the PXRD results in Figures S3‐S5. Based on these results, we also conducted the multi‐factor study focusing on the synergistic effect between the Si and SDA content. It should be noticed that the diffraction pattern difference between SAPO‐34 and SAPO‐18 is relatively small, and the intensity of the AEI‐characteristic shoulder peak at 10.4° 2θ is usually determined by the size of the AEI domain and the CHA/AEI arrangement in the intergrowth. Thus, we only exhibit the fraction of AFI and CHA (also including CHA/AEI intergrowth) lattices in this multi‐factor study, as given in Figure 2b. Semi‐quantitative analysis of the fraction of SAPO‐5 (AFI) and SAPO‐34/18 (CHA/AEI) was performed using the reference intensity method, with a known amount of α‐Al_2_O_3_ added as an internal standard. In Figure 2b, we integrated the influence of Si/Al ratio, total SDA concentration, and TEA/TEAOH ratio on the SAPO zeolite composition into a four‐dimensional diagram. The obvious color difference used to distinguish between SAPO‐34/18 and SAPO‐5 clearly describes two crystallization regimes: (i) the optimal window for the SAPO‐34/18 intergrowth at moderate Si/Al ratios (0.08–0.12), higher total SDA concentrations, and lower TEA fractions, and (ii) the optimal window favoring the SAPO‐5 formation at lower Si/Al ratios (< 0.08), lower total SDA concentrations, and higher TEA fractions.

In the separated plots extracted from Figure 2b, the influence law of these parameters can also be clearly observed (Figure 2c–h and Figure S6). For example, comparing Figure 2c–e, we find that the synthesis window for high SAPO‐5 content is predominantly localized at lower Si/Al ratios and higher total SDA concentrations. The synergistic effect of these two parameters just reflects the competition between thermodynamic and kinetic preferences. In the absence of SDAs or at very low SDA concentrations, the SAPO‐5 exhibits higher thermodynamic stability compared to the SAPO‐34, resulting in the main product being hexagonal prismatic SAPO‐5 particles. Due to the function of SDAs, the SAPO zeolite will kinetically form the SAPO‐34 or SAPO‐18 structures that match the size and topology of SDAs. The doping of Si atoms can make the local framework negatively charged, thereby tending to bind positively charged SDAs (TEA^+^ form) and ultimately achieve the result of enriching SDAs. Meanwhile, Figure 2f–h also exhibits the influence of TEA/TEAOH ratio, which actually has a similar explanation to the total SDA concentration. On the one hand, compared to TEAOH, TEA has one less ethyl branch, making its structure‐directing function a little weaker. On the other hand, TEA needs to be hydrolyzed to exert its function, so an increase in TEA may reduce the true concentration of SDAs in the solution. Through a series of explorations, from single‐factor to multi‐factor studies, we successfully deconvoluted the complex multivariable effects that govern topology selectivity during the hydrothermal synthesis of SAPO zeolites, establishing a foundation for the precise and controllable synthesis of SAPO catalysts with targeted topology composition and architecture.

### 2.3 Atomic Imaging of SAPO‐34 and SAPO‐18 Lattices

**FIGURE 3:**
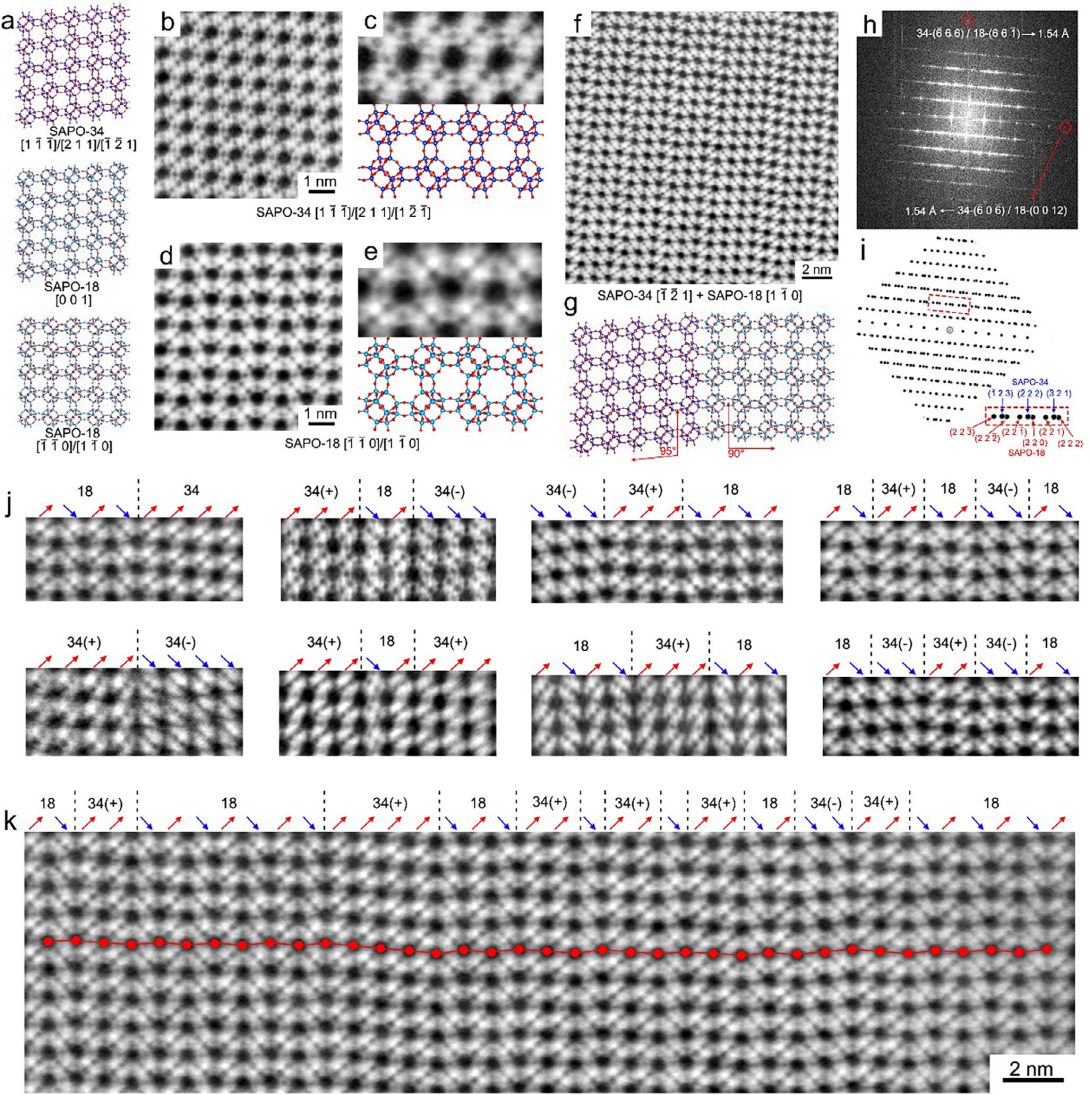
Atomically resolving the SAPO‐34/18 intergrowths. (a) Structural models of the CHA and AEI frameworks viewed along different crystallographic axes. (b, c) Magnified iDPC‐STEM images of the SAPO‐34 lattice from the given projection. (d, e) Magnified iDPC‐STEM images of the SAPO‐18 lattice from the given projection. (f–i) Coexisting SAPO‐34 and SAPO‐18 lattices in their intergrowth confirmed by the iDPC‐STEM image and corresponding FFT pattern (information transfer of 1.54 Å). (j, k) Different stacking sequences of layered units observed in the SAPO‐34/18 intergrowths, displaying the spatial distribution of mixed SAPO‐34 and SAPO‐18 lattices.

Although we can use the PXRD to distinguish between SAPO‐34 (or SAPO‐18) and SAPO‐5, two structures with large lattice differences, and their controlled synthesis conditions, it is still very difficult to accurately identify the lattice structures of SAPO‐34 and SAPO‐18 [21], as well as their composition and distribution in their intergrowths. The lack of in‐depth structural characterization and atomic insight into the SAPO‐34/18 intergrowth has prevented the establishment of a clear, quantitative correlation between synthesis parameters and the resultant framework architecture. To overcome the challenges posed by the electron‐beam sensitivity of zeolites and the low contrast of framework light elements, we employed iDPC‐STEM, which enables high‐resolution imaging of beam‐sensitive materials while preserving structural integrity. Figure 3a displays the structural models of SAPO‐34 (CHA framework) and SAPO‐18 (AEI framework) in distinct crystallographic projections. In these models, blue and cyan spheres represent T‐atoms (Si, Al, or P) occupying tetrahedral sites, while red spheres denote oxygen atoms. Notably, the projected atomic arrangement of the CHA framework along three orthogonal directions are crystallographically equivalent to that of AEI framework along the [001] direction. However, viewed in the [−1−10] or [1−10] projections of AEI framework, the layered units exhibit an alternating stacking sequence as we discussed in Figure 1. Remarkably, the imaged stacking sequence in SAPO‐34 can be observed in the magnified iDPC‐STEM images in Figure 3b,c, which is in perfect agreement with the model of CHA framework shown in Figure 3a. Similarly, atomic imaging of SAPO‐18 in Figure 3d and e reveals its fine structure that is fully consistent with the model of AEI framework.

Leveraging this atomic‐level imaging capability, we can determine the composition of the SAPO‐34/18 intergrowth in real space by directly analyzing the stacking sequence of layered units and the orientation of channel systems. High‐resolution iDPC‐STEM image of the SAPO‐34/18 intergrowth in Figure 3f directly shows an alternating sequence of two distinct stacking modes (defined in Figure 1), corresponding to the coexistence of CHA and AEI domains within a single crystalline architecture, as shown in the structural model in Figure 3g. Reciprocal‐space analysis via the fast Fourier transform (FFT) of the intergrowth image in Figure 3f indicates an information transfer of 1.54 Å (Figure 3h and Figures S7 and S8), where the diffraction spots in the FFT pattern are elongated into streaks due to the overlapping of two sets of diffraction patterns of SAPO‐34 and SAPO‐18, respectively. The simulated diffraction pattern in Figure 3i confirmed such observation, corroborating the alternating CHA/AEI arrangement and their intergrowth. Atomic iDPC‐STEM imaging directly reveals alternating stacking sequences of CHA and AEI layered units within single nanocrystals, rather than distinct phase‐separated regions. Correspondingly, the characteristic elongation of diffraction spots into streaks in the FFT patterns confirms the structural superposition of both phases within the same crystal instead of a physical mixture.

Based on the above analysis of intergrowth structures and stacking modes by the iDPC‐STEM imaging, we can systematically identify a variety of distinct stacking sequences within the SAPO‐34/18 intergrowth using the combinations of SAPO‐34(+), SAPO‐34(−), and SAPO‐18 domains as defined in Figure 1. Representative examples of these stacking sequences are illustrated in Figure 3j, including various segments consisting of 18–34(+), 34(+)‐18‐34(−), 34(−)‐34(+)‐18, 18–34(+)‐18‐34(−)‐18, 34(+)‐34(−), 34(+)‐18‐34(+), 18–34(+)‐18, and 18–34(−)‐34(+)‐34(−)‐18. Such diverse stacking segments give rise to complex pore (cage) arrangement and channel direction, substantially enriching the microporous structural landscape of the SAPO‐34/18 intergrowth. Given that micro‐scale single zeolite crystal will consist of hundreds to thousands of such layered units, the theoretical number of accessible stacking sequences within intergrowth systems is essentially limitless, thereby providing extensive potential for the tailored microstructures of intergrowths. For example, we acquired an iDPC‐STEM image over a larger region of the SAPO‐34/18 intergrowth sample (Figure 3k), where we are able to unambiguously resolve an extraordinarily complex stacking sequence representative of alternating CHA and AEI domains. By tracing and connecting red dots in the image, the direction of pores and channels resulting from this particular stacking sequence can be clearly marked out. The observations based on advanced characterization technique reveal a great diversity in the stacking sequences and pore structures during the assembly of CHA and AEI lattices.

### 2.4 Resolving and Controlling SAPO‐34/18 Intergrowths

**FIGURE 4:**
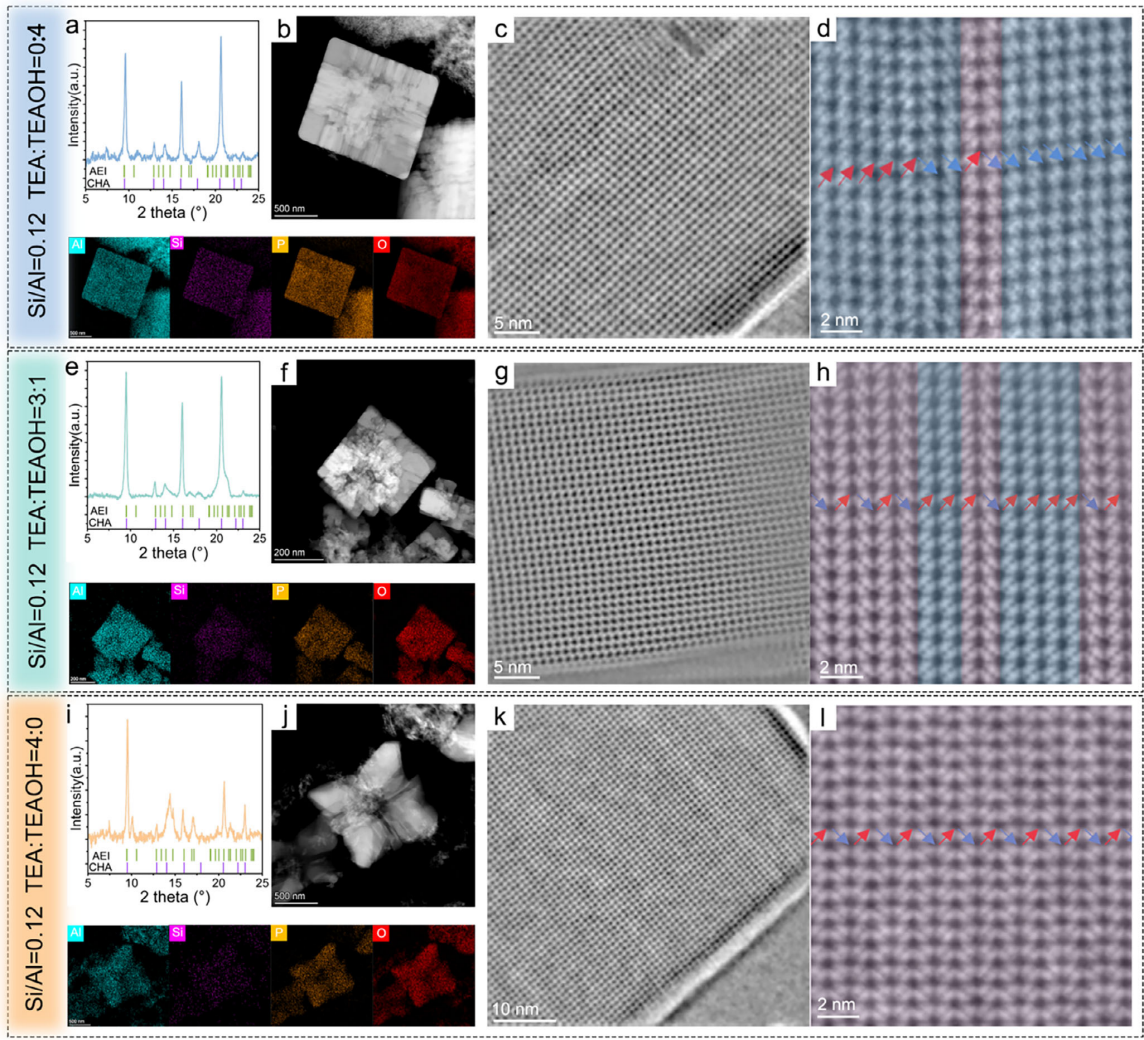
Structural control and characterization of the tunable SAPO‐34/18 intergrowths. (a–d) PXRD (a), HAADF‐STEM image and elemental analysis (b), and iDPC‐STEM images (c, d) of the SAPO‐34/18 intergrowth sample using TEAOH as SDA (named SP‐2). (e–h) PXRD (e), HAADF‐STEM image and elemental analysis (f), and iDPC‐STEM images (g, h) of the SAPO‐34/18 intergrowth sample using TEA and TEAOH (3:1) as SDAs (named SP‐3). (i–l) PXRD (i), HAADF‐STEM image and elemental analysis (j), as well as iDPC‐STEM images (k, l) of the SAPO‐34/18 intergrowth sample using TEA as SDA (named SP‐4).

Then, combining the iDPC‐STEM with other characterization techniques (Figures S9–S11), we chose the parameter of TEA/TEAOH ratio as an example to explore the controllable synthesis of SAPO‐34/18 intergrowths. When employing TEAOH as the sole SDA, the PXRD pattern in Figure 4a confirms the formation of SAPO‐34 dominated structure since there is nearly no observed shoulder peak at 10.4° and 21° 2θ. The HAADF‐STEM image and corresponding elemental mapping in Figure 4b show its regular cubic shape and elemental composition. Consistent with the solid crystal morphology, the N_2_ adsorption‐desorption analysis confirms its predominantly microporous nature with a Brunauer‐Emmett‐Teller (BET) surface area of 383 m^2^/g (Figure S12). The iDPC‐STEM images in Figure 4c,d further help to resolve the lattice structure inside this sample, indicating the predominance of the CHA framework with few AEI segments as stacking faults. After replacing partial TEAOH with TEA (TEA:TEAOH = 3:1), the shoulder peaks at 10.4° and 21° 2θ in the PXRD pattern can be vaguely observed, indicating that continuous AEI segments have appeared in this sample (Figure 4e). The HAADF‐STEM image of this sample demonstrates a gradual hollowing of the cubic crystal morphology extending radially from the crystal surface to the interior (Figure 4f). In agreement with this structural hollowing, its N_2_ adsorption‐desorption isotherm reveals the appearance of a small amount of mesopores alongside a measured BET surface area of 356 m^2^/g (Figure S13). The iDPC‐STEM images in Figure 4g,h confirm the coexistence of CHA and AEI domains with approximately equal proportions. Notably, nanoscale AEI domains are difficult to identify by these shoulder peaks in PXRD, highlighting the critical importance of real‐space imaging for accurate structure assignment in intergrowth structures.

Using pure TEA as SDA, the PXRD pattern exhibits more reflections of the AEI lattice (shown by the separate peaks at 10.4° and 21° 2θ in Figure 4i), and the crystal morphology turns to irregular hollow particles (Figure 4j and Figure S14). Correspondingly, N_2_ adsorption‐desorption isotherm demonstrates the generation of abundant mesopores and the emergence of macropores within these irregular hollow particles, leading to a significantly increased BET surface area of 699 m^2^/g (Figure S15). The iDPC‐STEM images in Figures 4k and l indicate that most of the lattice regions conform to the stacking sequence of AEI framework. To better elucidate the phase distribution trends of CHA and AEI, we conducted a statistical analysis of high‐resolution iDPC‐STEM images (including Figure 4d,h,l) to estimate the approximate proportion of CHA/AEI domains. The results showed that, as the TEA ratio increased, the proportion of AEI rose steadily from ∼10% to ∼50%, eventually reaching ∼90% or higher. Comparing the elemental analysis in Figure 4b,j, the obvious difference in Si content in the final product (although the Si/Al ratios in initial gels are the same) can help us explain such intergrowth structure change. The TEA^+^ ions can enhance the stability of Si‐doped lattice fragments by stabilizing the negatively charged framework, thereby increasing the Si content in the product, which kinetically supports the formation of CHA structure with higher symmetry. On the contrary, in the areas with lower TEAOH density, layered units stack in a freer manner, forming the AEI domains represented by an irregular sequence. By integrating PXRD, elemental analysis, and multiscale imaging results, this comprehensive strategy not only advances our understanding of how synthesis parameters govern the atomic local structure and macroscopic morphology of SAPO zeolites but also establishes a methodological basis for the rational design of zeolite frameworks with tailored microporous structures and optimized catalytic performance.

## 3 Conclusion

In summary, we have provided an in‐depth investigation into the multi‐scale characterization and controllable synthesis of SAPO zeolites. Focusing on a series of zeolite structures, including SAPO‐34/18 intergrowths and SAPO‐5 mixtures, we systematically explored the effects of various typical synthesis conditions and parameters, such as pH, Si content, SDA content, and dual SDA ratio, on the resultant structure distribution. Using the PXRD method, the relative proportions of SAPO‐34/18 (considered as a single phase for analysis) and SAPO‐5 can be clearly distinguished via their characteristic diffraction peaks, allowing us to analyze the preference of the SAPO‐34/18 and SAPO‐5 synthesis under different parameter windows. We identified a favorable synthesis window that enables the selective formation of SAPO‐34/18 intergrowths without SAPO‐5 impurities, and the underlying mechanism of such selectivity was interpreted in terms of competitive thermodynamic and kinetic preferences. Furthermore, for the SAPO‐34/SAPO‐18 intergrowth structures, which are difficult to resolve by conventional PXRD, we employed low‐dose iDPC‐STEM imaging to achieve atomic‐scale structural visualization of these beam‐sensitive zeolite materials, particularly the nanosized alternating SAPO‐34 and SAPO‐18 domains. The observed diversity in the layered units and their stacking sequences provided an unprecedented perspective into the assembly of zeolite intergrowths. On this basis, we further revealed the influence of dual SDA ratio on the composition and spatial distribution within SAPO‐34/18 intergrowths. The research methodology and successful research case presented in this work illustrate a blueprint for the controllable synthesis of targeted zeolite architectures with tailored microporous properties for advanced catalysis.

## Conflicts of Interest

The authors declare no conflicts of interest.

## Supporting information




**Supporting File**: advs76192‐sup‐0001‐SuppMat.docx.

## Data Availability

The data that support the findings of this study are available from the corresponding authors upon request.
