# Supplementary material for: Resolving and Controlling Silicoaluminophosphate Zeolite Intergrowths and Mixtures
Source: Adv Sci (Weinh). 2026 Jun 19:e76192. Online ahead of print. doi: 10.1002/advs.76192 (PMC13336472; doi:10.1002/advs.76192)
Supplement: Supplementary file 1 — Supporting File: advs76192‐sup‐0001‐SuppMat.docx. [file ADVS-9999-e76192-s001.docx]

**Supporting information**

**Resolving and Controlling Silicoaluminophosphate Zeolite** **Intergrowths and Mixtures**

*Yuxin Ke^1,2†^, Jiale Feng^1,2†^, Lei Wang^1,2†^, Xiao Chen^3^, Mengmeng Ma^1,2^, Jiabin Cui^4^, Juan Diwu^4^, Yuman Liu^5*^, Fei Wei^3^, Boyuan Shen^1,2^*, Bin Song^1,2^**

**Affiliations:**

^1^ Institute of Functional Nano & Soft Materials (FUNSOM), Soochow University, Suzhou, 215123, Jiangsu, PR China.

^2^ Jiangsu Key Laboratory of Advanced Negative Carbon Technologies, Soochow University, Suzhou, 215123, Jiangsu, PR China

^3^ Beijing Key Laboratory of Green Chemical Reaction Engineering and Technology, Department of Chemical Engineering, Tsinghua University, Beijing 100084, PR China.

^4^ State Key Laboratory of Radiation Medicine and Protection, School of Radiation Medicine and Protection, Collaborative Innovation Center of Radiological Medicine of Jiangsu Higher Education Institutions, Soochow University, Suzhou 215123, Jiangsu, PR China

^5^ Suzhou National Laboratory, Suzhou, 215000, Jiangsu, PR China.

^#^ These authors contributed equally to this work.

* Corresponding authors. Emails: bsong@suda.edu.cn (B. Song); liuym@szlab.ac.cn (Y. Liu); byshen@suda.edu.cn (B. Shen)

**Contents**

**1. Materials and Methods**

1.1 Materials

1.2 Synthesis method of SAPO-n zeolites

1.3 Characterization

**2. Supplementary Figures and Tables**

Table S1. Synthesis ratios for several typical SAPO-n zeolites samples.

Figure S1. SEM and TEM images of SAPO-5 (sample named SP-1)，clearly revealing a hexagonal plate-like structure.

Figure S2. N_2_ adsorption-desorption isotherms and pore size distribution of SAPO-5 (sample named SP-1).

Figure S3. PXRD results of SAPO-34/18 intergrowth and SAPO-5 synthesized with different Si/Al ratio in feed for single condition.

Figure S4. PXRD results of SAPO-34/18 intergrowth and SAPO-5 synthesized with different SDA(TEA+TEAOH) content in feed for single condition.

Figure S5. PXRD results of SAPO-34/18 and SAPO-5 intergrowth synthesized with different TEA/TEAOH ratio in feed for single condition.

Figure S6. PXRD Semi-quantitative results of SAPO-34/18 intergrowth and SAPO-5 synthesized between Si/Al ratio and more SDA (TEA+TEAOH) content or different TEA/TEAOH ratios for Two-factor conditions.

Figure S7. FFT pattern of SAPO-34 from Figure 3b iDPC-STEM image (information transfer of 1.54 Å).

Figure S8. FFT pattern of SAPO-18 from Figure 3d iDPC-STEM image (information transfer of 1.68 Å).

Figure S9. SEM and TEM images of SAPO-34 (using TEAOH as the template agent，sample named SP-2).

Figure S10. SEM and TEM images of SAPO-34/18 synthesized with TEA and TEAOH (molar ratio =3:1，sample named SP-3).

Figure S11. SEM and TEM images of mainly SAPO-18 only synthesized with TEA (sample named SP-4).

Figure S12. N_2_ adsorption-desorption isotherms and pore size distribution of SAPO-34 (using TEAOH as the template agent，sample named SP-2).

Figure S13. N_2_ adsorption-desorption isotherms and pore size Distribution of SAPO-34/18 synthesized with TEA and TEAOH (molar ratio =3:1，sample named SP-3).

Figure S14. PXRD patterns illustrating the storage stability of the SAPO-34/18 intergrowth sample (SP-3) synthesized using TEA and TEAOH (3:1) over a 6-month period at room temperature.

Figure S15. N_2_ adsorption-desorption isotherms and pore size distribution of SAPO-18 only synthesized with TEA (sample named SP-4).

1. **Materials and Methods**
   1. *Materials*

The following materials were used as received: pseudoboehmite (AlOOH·nH_2_O,n= 0. 08~0.62, Macklin, ＞99.0%) as an aluminum source, Tetraethoxysilane (TEOS, Adamas, 99%) as a silica source, Phosphoric acid(H_3_PO_4_,Greagent,85% in water) as Phosphorus source, Tetraethylammonium Hydroxide (TEAOH,Adamas,25% in water) and Triethylamine (TEA, Greagent, ＞99.0%) as the organic structure-directing agent. The pH is adjusted using Hydrochloric acid (HCl,yonghua, ＞99.8%).

- 1. *Synthesis method of SAPO-n zeolites*

All zeolites were synthesized using hydrothermal methods in a sealed Teflon-lined Parr reactor. Brief details are provided below.

SAPO-n (n=5, 34/18) was synthesized by mixing pseudo boehmite (Al source), phosphoric acid (P source), tetraethyl orthosilicate (Si source), triethylamine (TEA, templating agent), tetraethyl ammonium hydroxide(TEAOH, templating agent), and deionized water to make a gel for stirring 2 h with the ratio:1 Al_2_O_3_ : xSiO_2_ : 0.9P_2_O_5_: yTEA: zTEAOH: 50H_2_O.In this work, the Si contents in feed are x=0.02-0.12, the total SDA(y+z) concentration (expressed as SDA/Al2O3, 0.7864-4.65), TEA(y): TEA+TEAOH(=y+z) ratios of 0, 0.25, 0.5, 0.75, 1 and initial gel pH (adjusted with hydrochloric acid to 5-8) were systematically varied. These samples also crystallized at 140 °C for 2 h, and then at 185 °C for another 24 h. After the same processes of filtration, washing, drying, and calcination at 550℃ for 5 h, we can obtain the powder samples under different synthesis conditions. It is worth noting that all pH values reported refer to the initial synthesis gel measured at room temperature before hydrothermal treatment. Although the pH undergoes changes during the hydrothermal process due to the dissolution and consumption of silicon, aluminium and phosphorus sources, as well as the template agent, the magnitude of these changes is relatively small. The initial pH still accurately reflects the acid-base environment of the crystallisation system and serves as a stable and reliable parameter for controlling the CHA/AEI phase ratio.

- 1. *Characterization*

Powder X-ray diffraction (XRD) patterns were obtained by a PANalytical diffractometer using Cu Kα radiation at 40 kV and 40 mA. The crystallinity was calculated on the basis of the areas of the peaks ranging from 5° to 50°.

The SEM images were captured by Zeiss G500 with an operating voltage of 5–20 kV.

The TEM of the sample was obtained by using the Talos F200X transmission electron microscope from FEI Corporation, with a test voltage of 200 kV.

The convergence semi-angle for the HAADF-STEM is 25 mrad. The collection angle for the HAADF-STEM is 5-27 mrad. The electron beam current for the HAADF-STEM is 80 pA. The dwell time for the HAADF-STEM is 2 μs/pixel. The EDS mapping was also conducted using the same aberration coefficients.

The beam current for the EDS is set between 50 and 100 pA to minimize the radiation damage to the specimens, and the dwell time is 2 µs/pixel with a map size of 256 × 256 pixels. A complete process of EDS mapping took roughly 10 min to reach enough SNR.

The N_2_ adsorption/desorption isotherms and corresponding Brunauer–Emmett–Teller surface areas of the samples were obtained by a surface area and porosity analyzer (CIQTEK V-Sorb 2800TP).

High-resolution iDPC-STEM imaging was performed using a Cs-corrected FEI Titan Cubed Themis G2 300 STEM operated at 300 kV and equipped with a DCOR+ spherical aberration corrector. The convergence semi-angle was set to 15 mrad, with a collection angle ranging from 3 to 18 mrad. The beam current was approximately 0.1 pA, as measured by a pixel array detector. Throughout each imaging session, the beam energy, electron dose rate, and detector gain were maintained constant to ensure comparability among datasets.

1. **Supplementary Figures and Tables**

| Sample | Al_2_O_3_:SiO_2_:P_2_O_5_:TEA:TEAOH:H_2_O | Composition |
| --- | --- | --- |
| SP-1 | 1:0.24:0.9:1.1168:0:36 | SAPO-5 |
| SP-2 | 1:0.24:0.9:0:1.966:50 | Mainly SAPO-34 |
| SP-3 | 1:0.24:0.9:1.4745:0.4915:50 | SAPO-34/18 |
| SP-4 | 1:0.24:0.9:1.966:0:50 | Mainly SAPO-18 |

**Table S1.** Synthesis ratios for several typical SAPO-n zeolites samples.


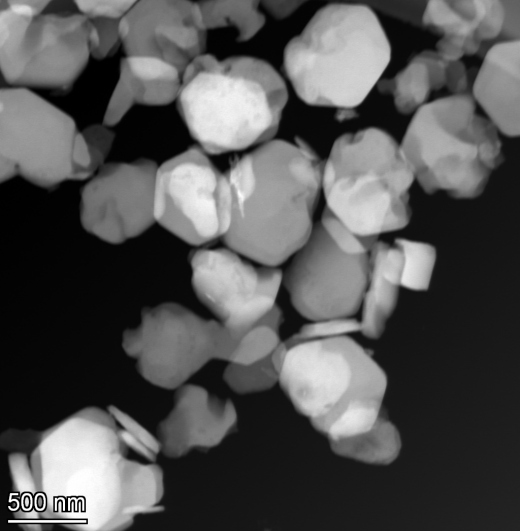

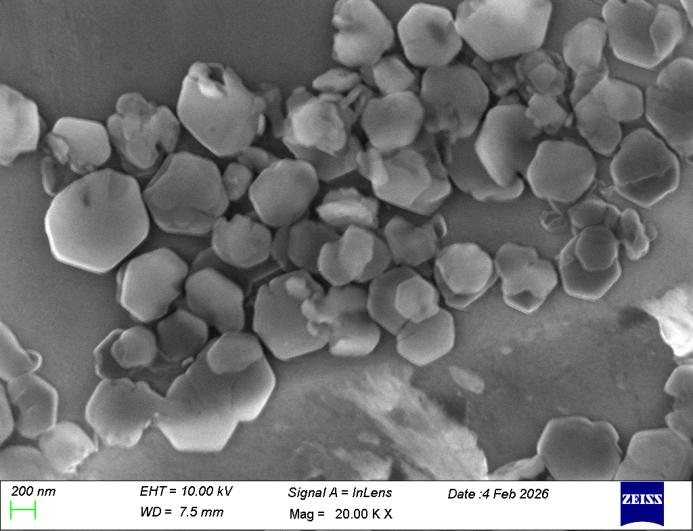

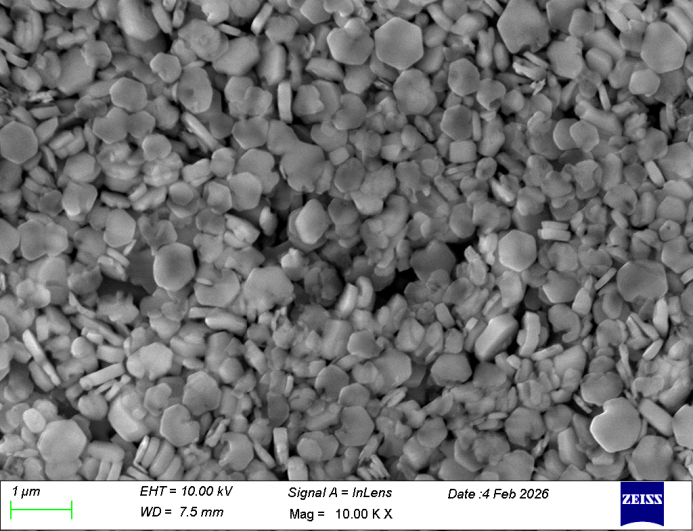

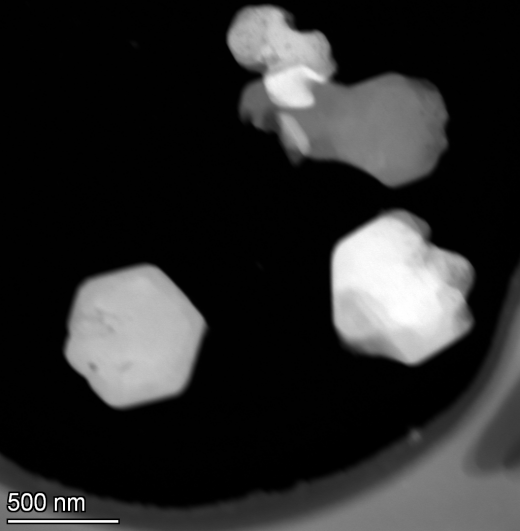


**Figure S1.** SEM and TEM images of SAPO-5 (sample named SP-1), clearly revealing a hexagonal plate-like structure.


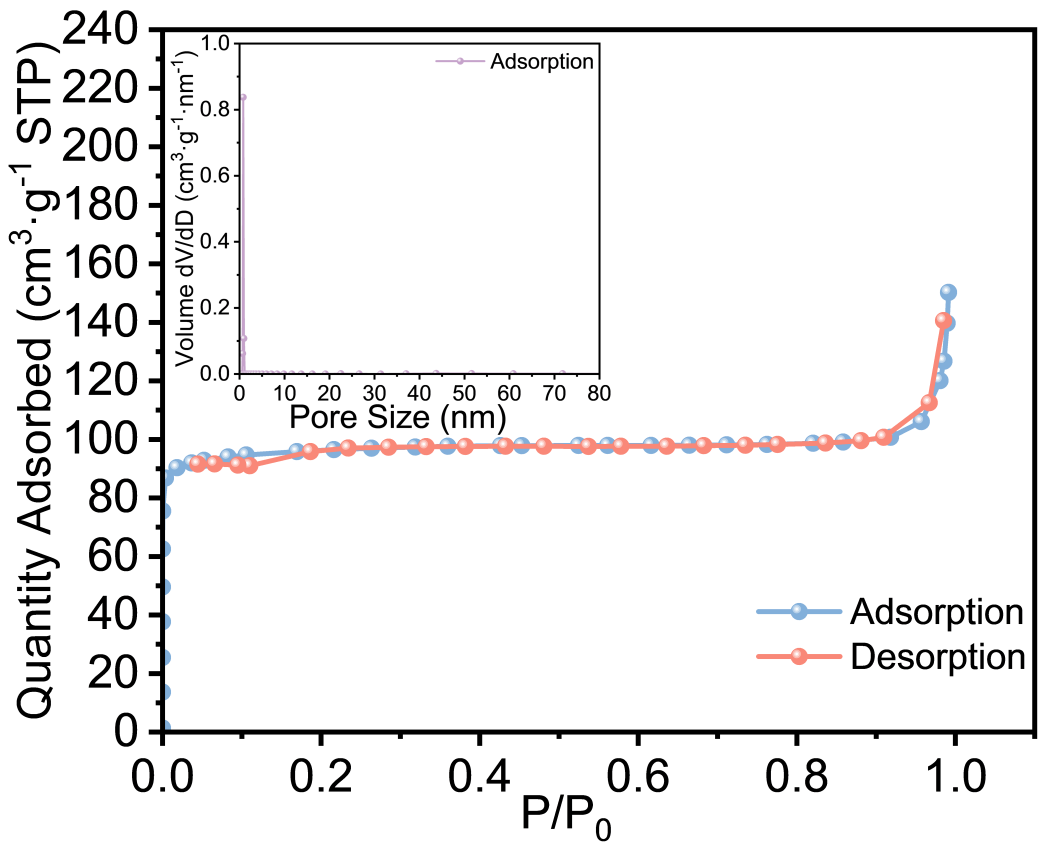


**Figure S2.** N_2_ adsorption–desorption isotherms and pore size distribution of SAPO-5 (sample named SP-1).


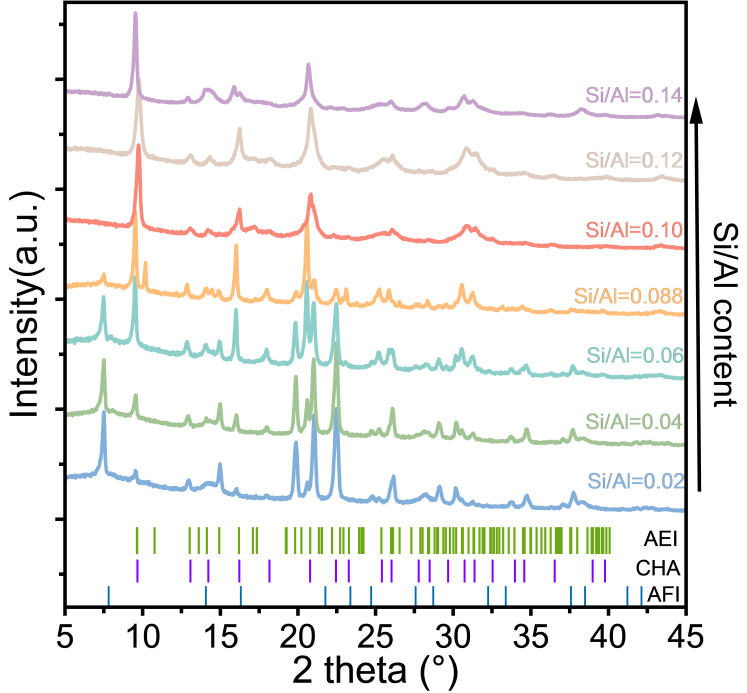


**Figure S3.** PXRD results of SAPO-34/18 intergrowth and SAPO-5 synthesized with different Si/Al ratio in feed for single condition.


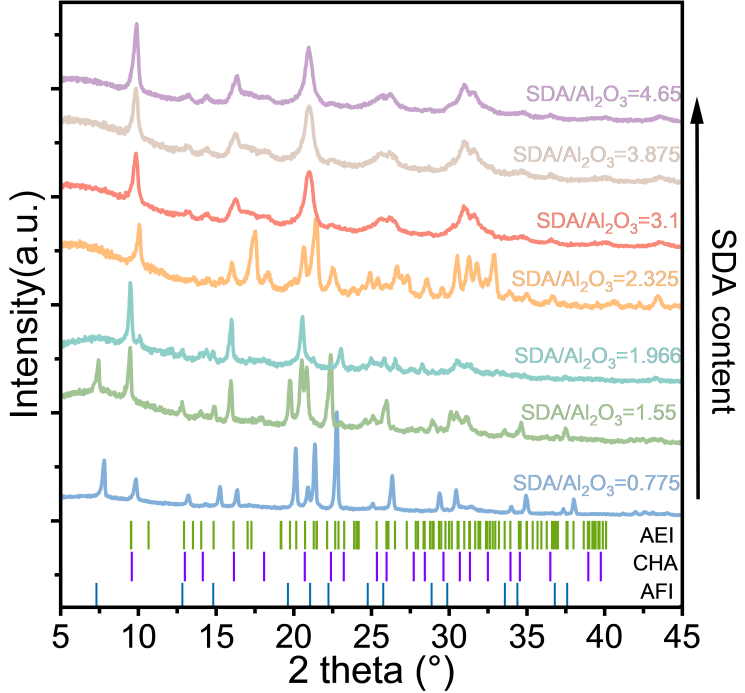


**Figure S4.** PXRD results of SAPO-34/18 intergrowth and SAPO-5 synthesized with different SDA(TEA+TEAOH) content in feed for single condition.


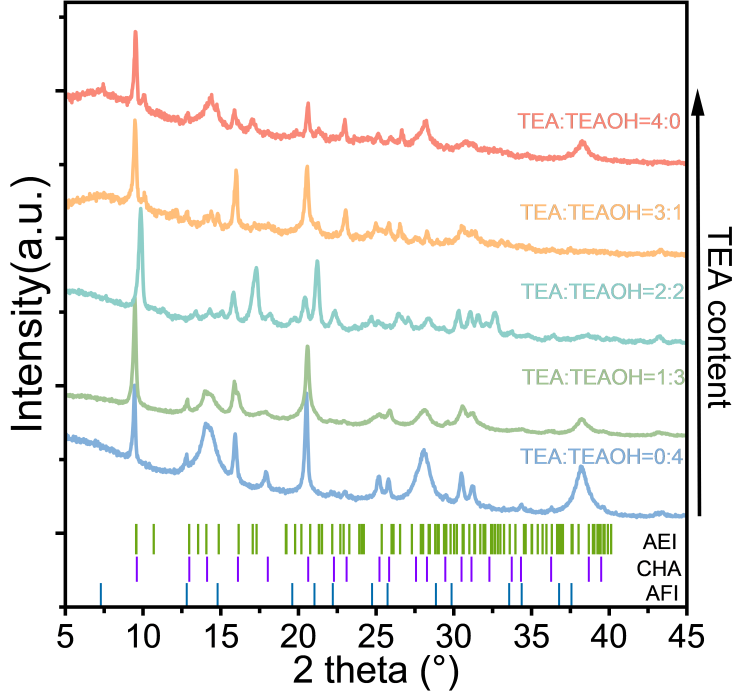


**Figure S5.** PXRD results of SAPO-34/18 and SAPO-5 intergrowth synthesized with different TEA/TEAOH ratio in feed for single condition.


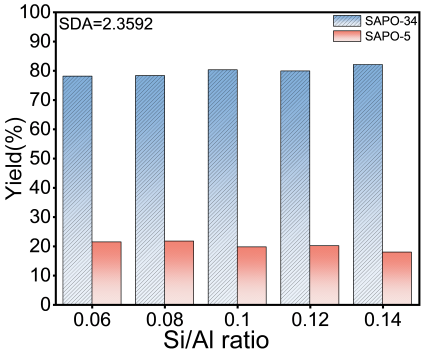

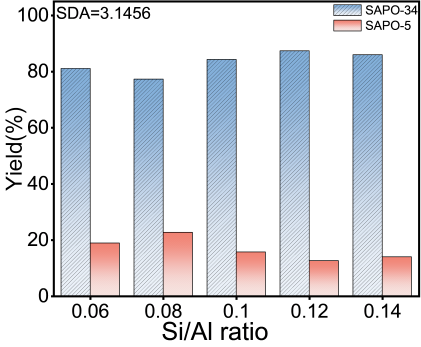

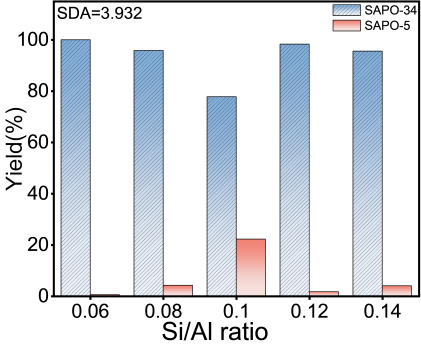


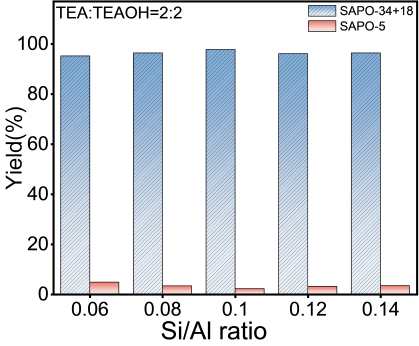

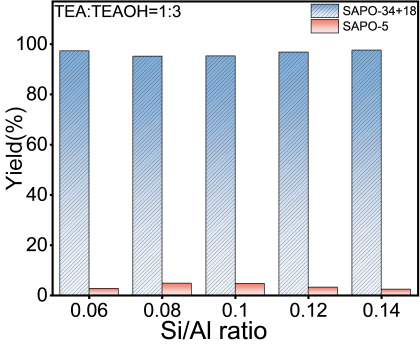


**Figure S6.** PXRD Semi-quantitative results of SAPO-34/18 intergrowth and SAPO-5 synthesized between Si/Al ratio and more SDA(TEA+TEAOH) content or different TEA/TEAOH ratios for Two-factor conditions.


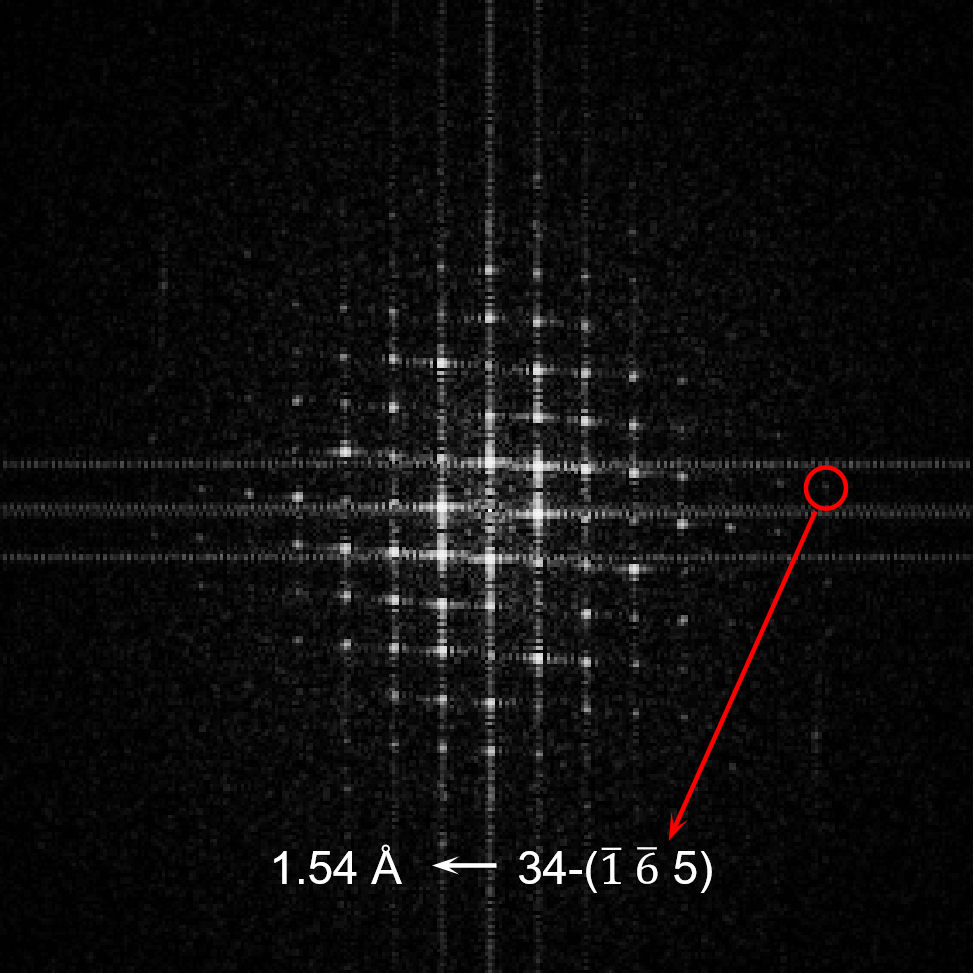


**Figure S7.** FFT pattern of SAPO-34 from Figure 3b iDPC-STEM image (information transfer of 1.54 Å).


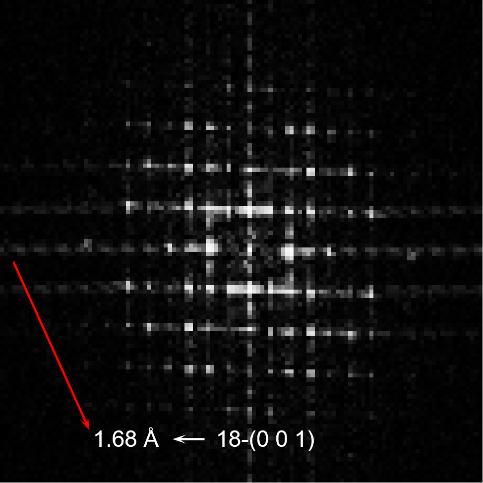


**Figure S8.** FFT pattern of SAPO-18 from Figure 3d iDPC-STEM image (information transfer of 1.68 Å).


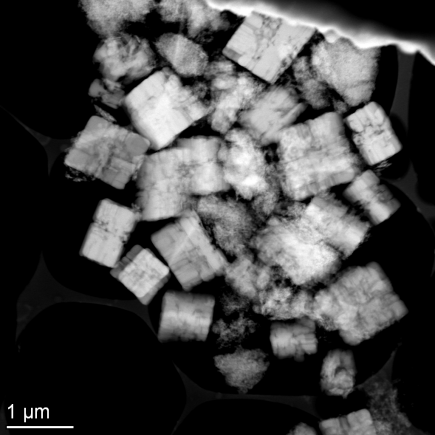

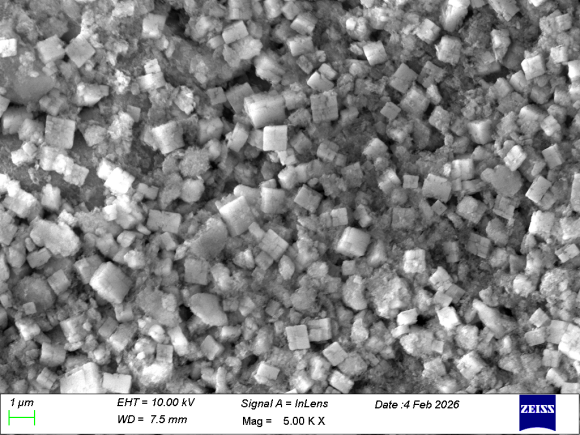

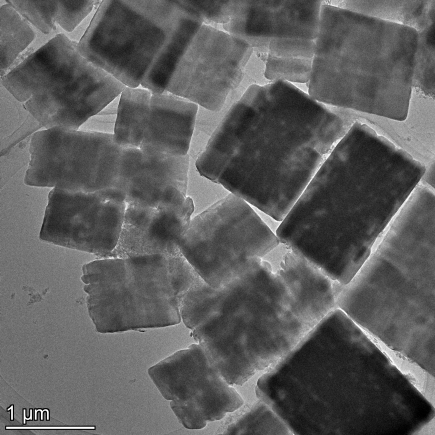

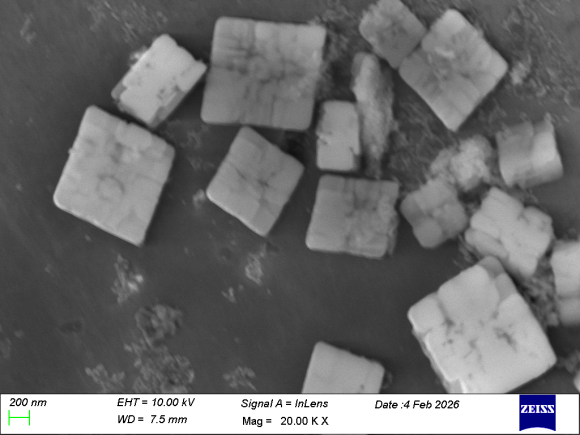


**Figure S9.** SEM and TEM images of SAPO-34 (using TEAOH as the template agent，sample named SP-2).


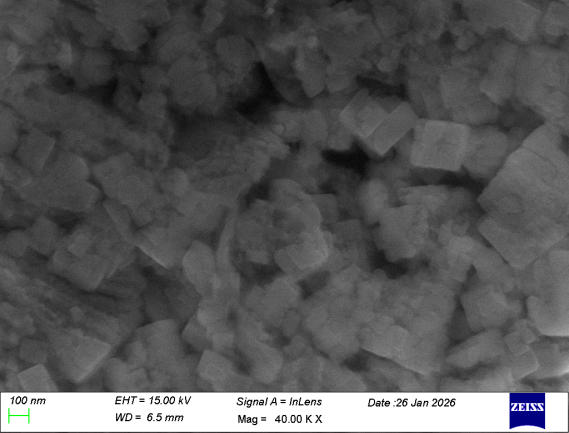

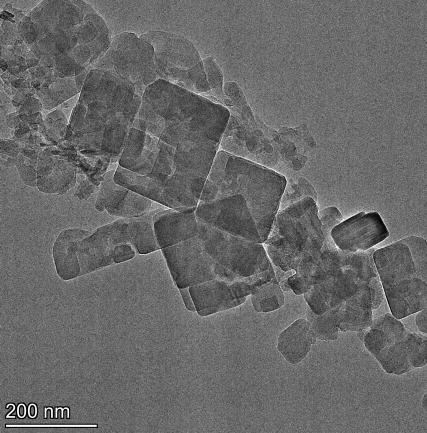

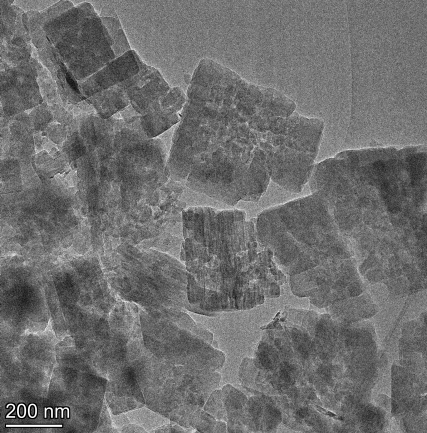

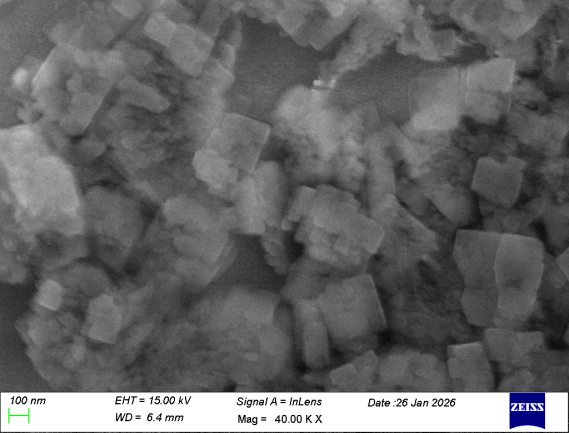


**Figure S10.** SEM and TEM images of SAPO-34/18 synthesized with TEA and TEAOH(molar ratio=3:1，sample named SP-3).


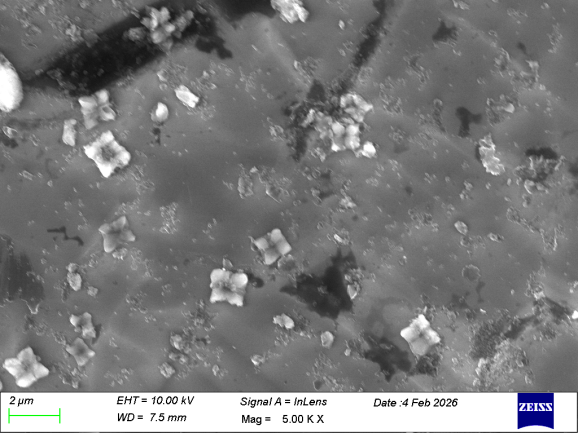

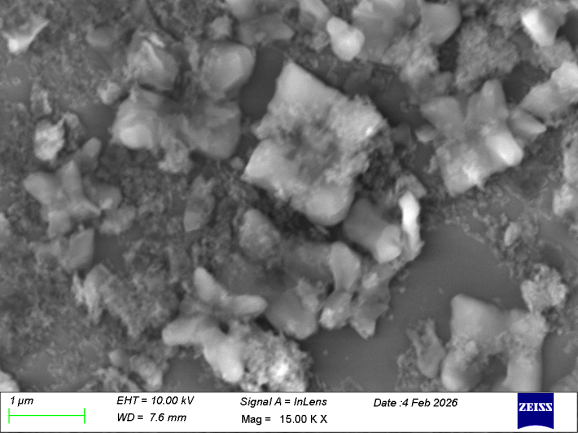

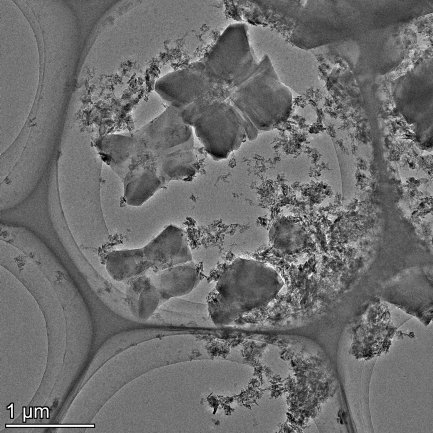

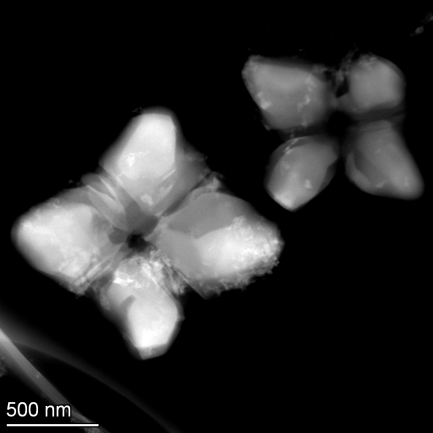


**Figure S11.** SEM and TEM images of mainly SAPO-18 only synthesized with TEA (sample named SP-4).


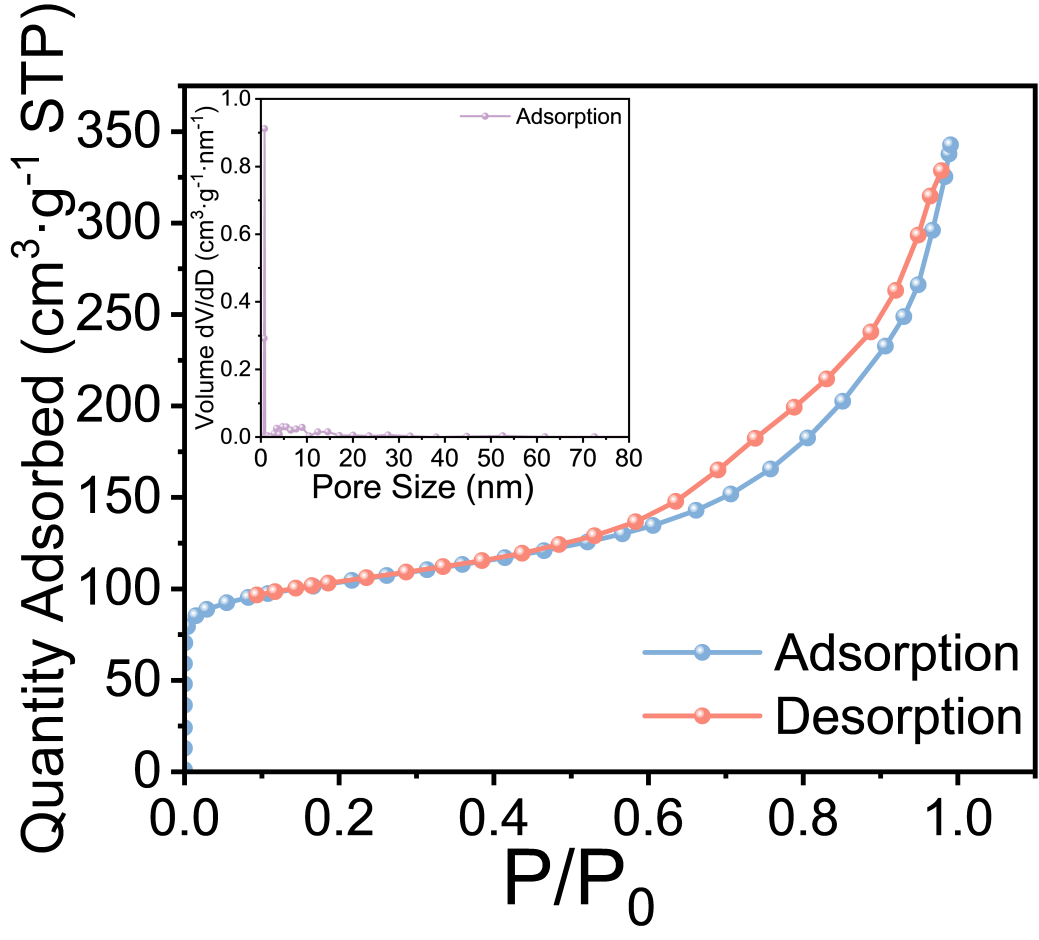


**Figure S12.** N_2_ adsorption–desorption isotherms and pore size distribution of SAPO-34 (using TEAOH as the template agent，sample named SP-2).


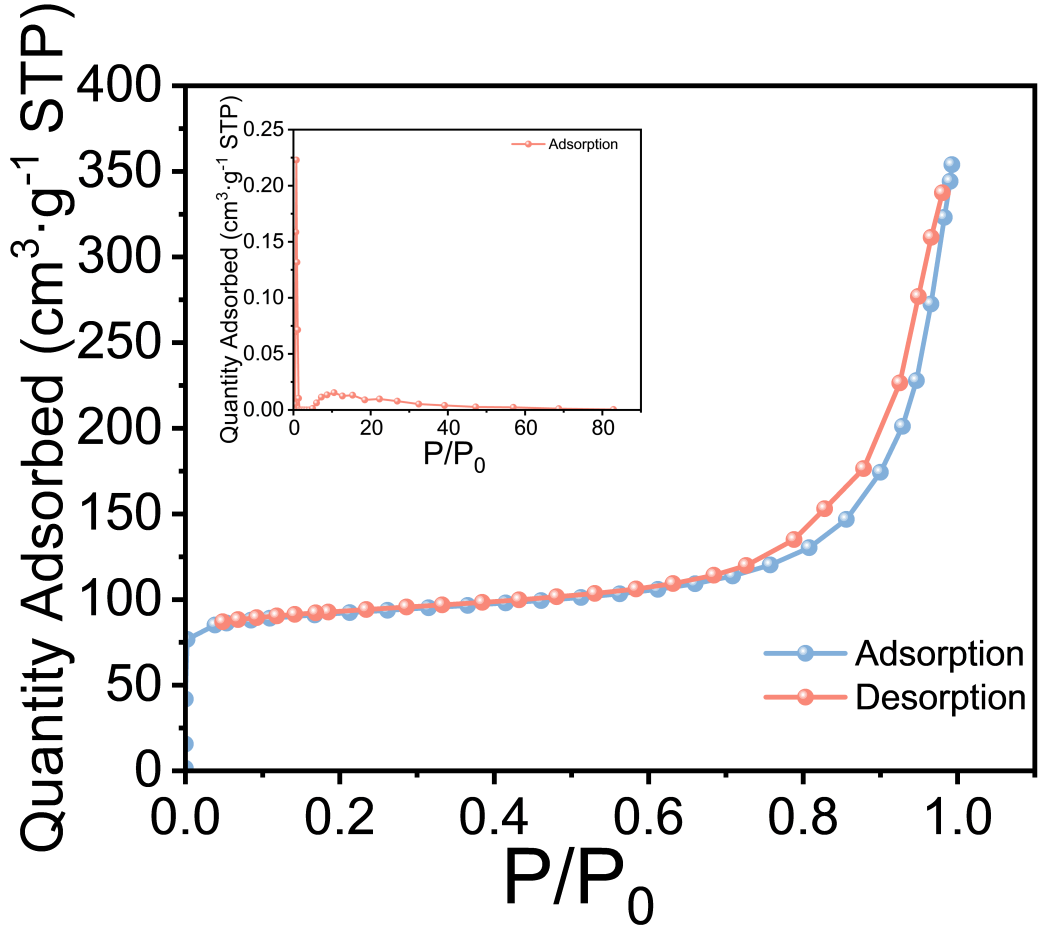


**Figure S13.** N_2_ adsorption–desorption isotherms and pore size distribution of SAPO-34/18 synthesized with TEA and TEAOH (molar ratio =3:1，sample named SP-3).


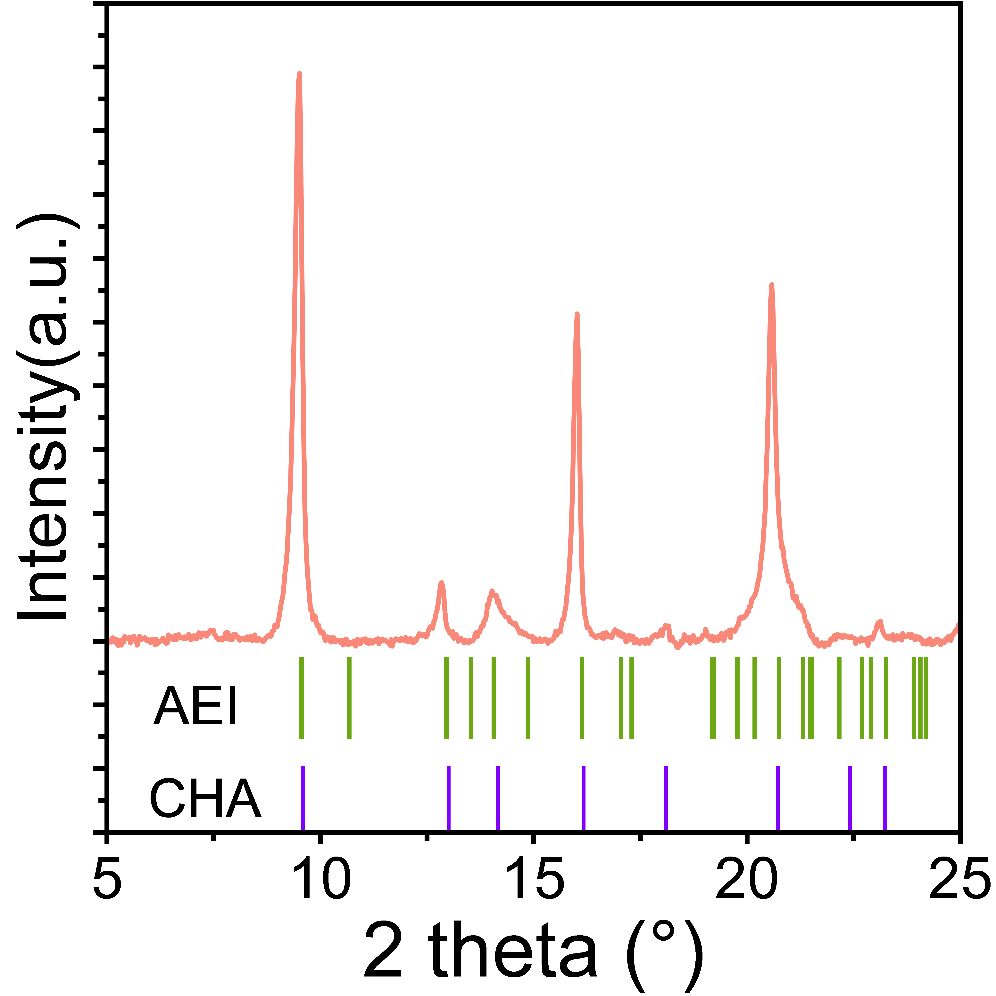


**Figure S14.** PXRD patterns illustrating the storage stability of the SAPO-34/18 intergrowth sample (SP-3) synthesized using TEA and TEAOH (3:1) over a 6-month period at room temperature.


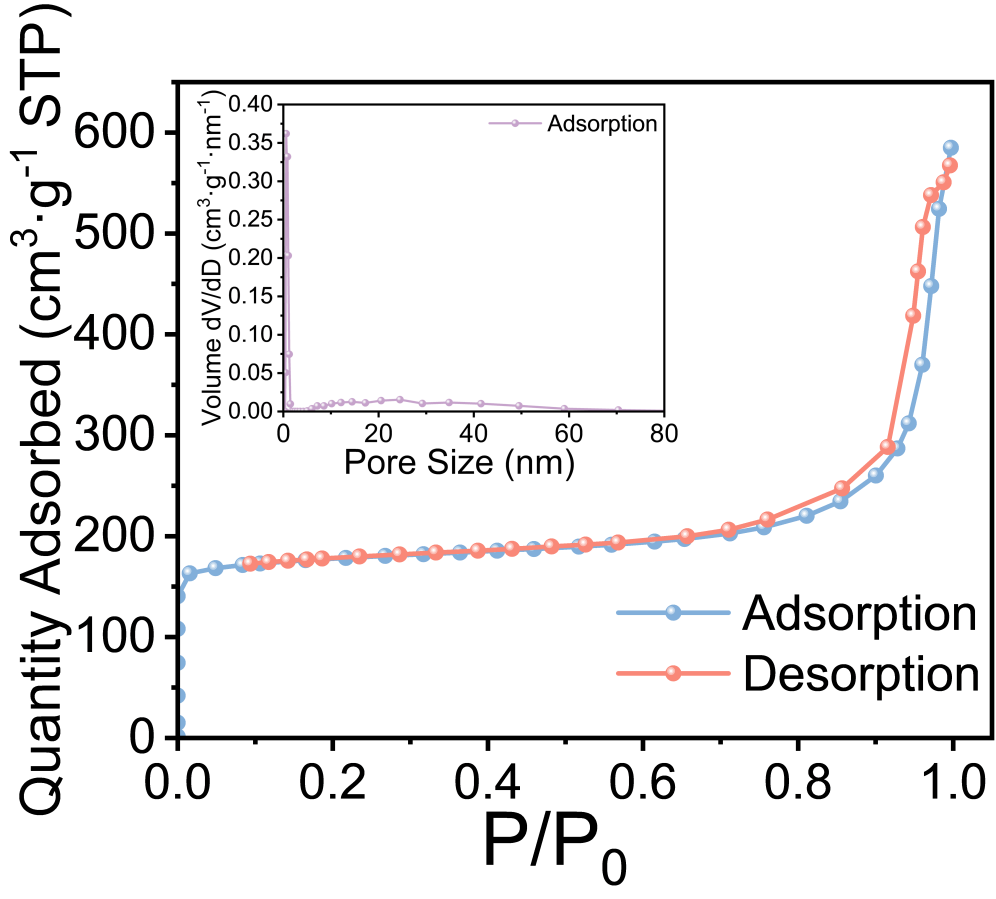


**Figure S15.** N_2_ adsorption–desorption isotherms and pore size distribution of SAPO-18 only synthesized with TEA (sample named SP-4).
